# PARTIAL REPROGRAMMING REVERSES FEATURES OF AGING IN HUMAN T LYMPHOCYTES

**DOI:** 10.1093/geroni/igae098.0869

**Published:** 2024-12-31

**Authors:** Jacob Kimmel

**Affiliations:** NewLimit, South San Francisco, California, United States

## Abstract

Youthful gene expression and function can be restored in old cells by brief activation of transcription factors (TFs), a process known as partial reprogramming. Partial reprogramming holds promise as a therapeutic approach, but current methods have only employed a small set of pluripotency TFs that also reprogram cell identity in undesirable ways. It remains unknown if other TFs might be able to reprogram cell age, how long these effects last, and how often TFs act in concert to promote youthful phenotypes. To address these questions, we developed a screening technology called Partial-seq to measure the effect of partial reprogramming on gene expression across thousands of TF sets at once. We used Partial-seq to screen >4000 TF sets in aged human T cells, a keystone cell type of the immune system that declines in function with age. We discovered that many TFs beyond pluripotency factors restored youthful gene expression and cell states, without abolishing T cell identity. TF combinations were more likely than individual factors to reverse cell age, and many of these effects persisted after TF activation ceased. Our results suggest that the phenotypic age of human T cells can be durably reversed by partial reprogramming with diverse TFs. The diversity of TF sets discovered in our screens suggests that reprogramming cell age might be accomplished without perturbing cell identity.

